# Neonatal gastroschisis associated with patent ductus arteriosus: a case report

**DOI:** 10.1093/jscr/rjag981

**Published:** 2026-10-27

**Authors:** Julio Caleb Arrieta Navarro, Daniel Villamizar Hernandez, Efrain A Morales Elescano, Karen J Huamán Guevara, Stefany M Marchena Ciudad

**Affiliations:** Faculty of Human Medicine, National University of San Martín, Tarapoto, Peru; Faculty of Human Medicine, National University of San Martín, Tarapoto, Peru; Department of Gynecology and Obstetrics, Obstetrics Service, Hospital II-2 Tarapoto, Tarapoto, Peru; Faculty of Human Medicine, National University of San Martín, Tarapoto, Peru; Department of Gynecology and Obstetrics, Obstetrics Service, Hospital II-2 Tarapoto, Tarapoto, Peru; Faculty of Human Medicine, National University of San Martín, Tarapoto, Peru; Department of Pediatrics, Neonatology Service, Hospital II-2 Tarapoto, Tarapoto, Peru; Faculty of Human Medicine, National University of San Martín, Tarapoto, Peru; Department of Pediatrics, Neonatology Service, Hospital II-2 Tarapoto, Tarapoto, Peru

**Keywords:** gastroschisis, patent ductus arteriosus, pediatric surgery, prenatal diagnosis, multidisciplinary management, prematurity

## Abstract

Gastroschisis is a congenital abdominal wall defect that requires early prenatal diagnosis, close fetal surveillance, appropriate delivery planning, and timely multidisciplinary management to improve neonatal outcomes. We present the case of a 23-year-old pregnant woman from Tarapoto, Peru, at 33 weeks and 3 days of gestation, who presented with decreased fetal movements. Specialized ultrasound revealed fetal gastroschisis and a true umbilical cord knot. Given evidence of fetal distress, an emergency cesarean section was performed. A preterm female infant weighing 1850 g was born with Apgar scores of 8 and 9 at 1 and 5 min, respectively. At birth, neonatal gastroschisis with exteriorization of intestinal loops was confirmed, requiring silo placement followed by delayed surgical closure. During hospitalization, the newborn developed patent ductus arteriosus, which was managed medically, as well as suspected neonatal sepsis and feeding difficulties. Coordinated management by obstetrics, neonatology, pediatric surgery, and pediatric cardiology allowed progressive clinical stabilization, intestinal adaptation, and advancement of enteral feeding. The patient had a favorable clinical course and was discharged at 28 days of age. This case highlights the importance of adequate prenatal care, recognition of warning signs such as decreased fetal movements, timely referral for specialized assessment, and multidisciplinary management of newborns with gastroschisis and associated complications. It also provides a practical learning point for healthcare professionals involved in the prenatal and neonatal care of these patients.

## Introduction

Gastroschisis is a congenital abdominal wall defect characterized by the herniation of bowel loops through a paraumbilical defect, usually located to the right of the umbilical cord insertion and lacking a protective membrane. Although relatively uncommon, its incidence has increased worldwide in recent decades, particularly among young mothers, making it one of the most relevant congenital gastrointestinal anomalies encountered in fetal medicine and neonatal surgery [1–4].

The condition is associated with significant neonatal morbidity due to prolonged exposure of the bowel to amniotic fluid, which may result in intestinal inflammation, edema, atresia, necrosis, sepsis, feeding difficulties, and prolonged hospitalization [1, 4]. Prenatal diagnosis through obstetric ultrasound plays a crucial role in the management of affected pregnancies by enabling early identification of the abdominal wall defect, appropriate fetal surveillance, delivery planning, and timely coordination of specialized neonatal and surgical care [5]. In this context, adequate prenatal counseling and education are also important, particularly to promote adherence to prenatal follow-up and prompt evaluation of warning signs such as decreased fetal movements.

A true umbilical cord knot is an uncommon condition that may be associated with fetal hypoxia, fetal distress, and adverse perinatal outcomes. When identified in a pregnancy complicated by gastroschisis, careful fetal surveillance and timely obstetric assessment are particularly important. In addition, preterm newborns with gastroschisis may develop complications such as patent ductus arteriosus (PDA), suspected infection, feeding intolerance, and respiratory or hemodynamic instability, which may further complicate their postoperative course [6]. Therefore, the management of these patients requires coordinated care involving obstetrics, neonatology, pediatric surgery, and, when necessary, pediatric cardiology.

We report the case of a preterm newborn with prenatal diagnosis of gastroschisis associated with a true umbilical cord knot who subsequently developed PDA and required surgical and medical management. The patient was successfully managed through a multidisciplinary approach at a regional hospital in the Peruvian Amazon. This case highlights the importance of adequate prenatal care, recognition of fetal warning signs, timely diagnosis and delivery planning, and coordinated multidisciplinary management throughout the neonatal course. It also provides practical educational considerations for healthcare professionals and families involved in the care of pregnancies and newborns affected by gastroschisis and associated complications.

## Case presentation

A 23-year-old pregnant woman from the Las Lomas de San Pedro sector of Tarapoto, Peru, with a history of miscarriage in 2021, was admitted to the emergency department of Hospital II-2 Tarapoto after experiencing a marked decrease in fetal movements for ~24–32 h. She had attended six prenatal checkups at a private healthcare facility. At admission, she was at 33 weeks and 2 days of gestation according to her last menstrual period and was hemodynamically stable, with a blood pressure of 123/71 mmHg and otherwise normal vital signs.

An initial obstetric ultrasound showed a “double bubble” sign, raising suspicion of intestinal atresia. A subsequent specialized maternal–fetal medicine ultrasound demonstrated a singleton pregnancy with a right-sided abdominal wall defect consistent with gastroschisis and an image suggestive of a true umbilical cord knot. Electronic fetal monitoring showed a non-reactive tracing with variable decelerations reaching 90 beats/min, consistent with fetal distress. Intestinal atresia was not subsequently identified during neonatal surgical exploration, indicating that the initial “double bubble” finding was not confirmed postnatally (Fig. 1).

**Figure 1:**
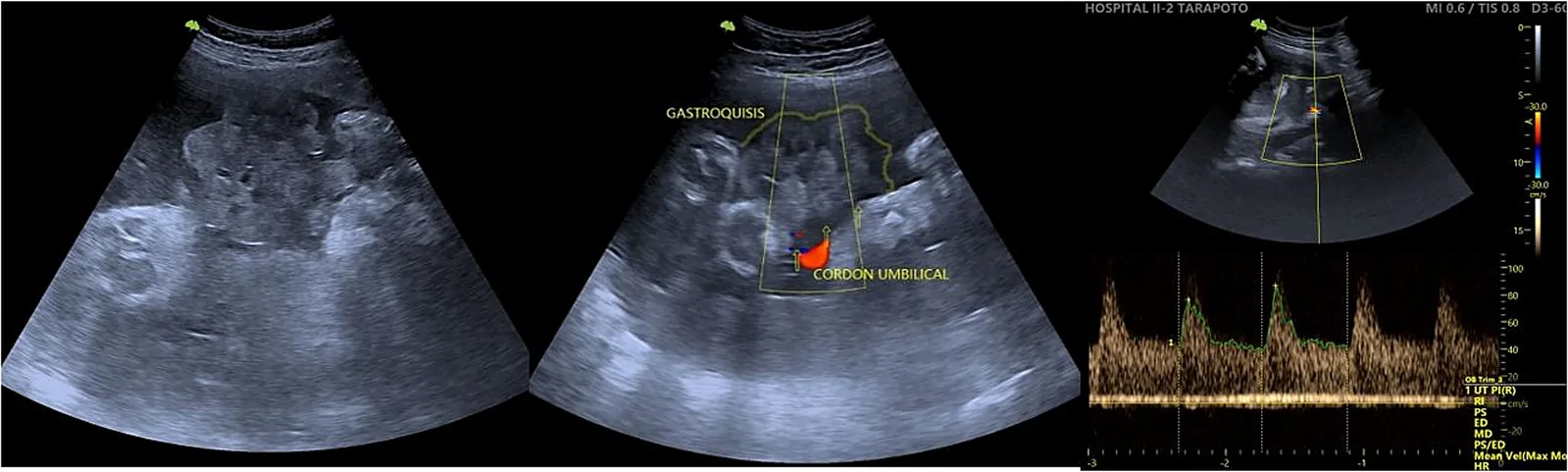
Prenatal ultrasound performed during the third trimester showing a paraumbilical abdominal wall defect with exteriorization of intestinal loops, findings consistent with fetal gastroschisis.

Because of the fetal findings, persistent uterine contractions despite tocolytic therapy, and evidence of fetal distress, a multidisciplinary team involving obstetrics, neonatology, and pediatric surgery evaluated the patient. An emergency transverse cesarean section was performed ~1 day after admission. Intraoperatively, gastroschisis and a true umbilical cord knot were confirmed.

A preterm female infant was delivered, weighing 1850 g, measuring 42 cm in length, and with a head circumference of 30 cm. Apgar scores were 8 and 9 at 1 and 5 min, respectively. Physical examination immediately after birth revealed a right paraumbilical abdominal wall defect with exteriorization of intestinal loops, confirming neonatal gastroschisis. The newborn was transferred to the Neonatal Intensive Care Unit for stabilization, respiratory support, and specialized surgical management (Fig. 2).

**Figure 2:**
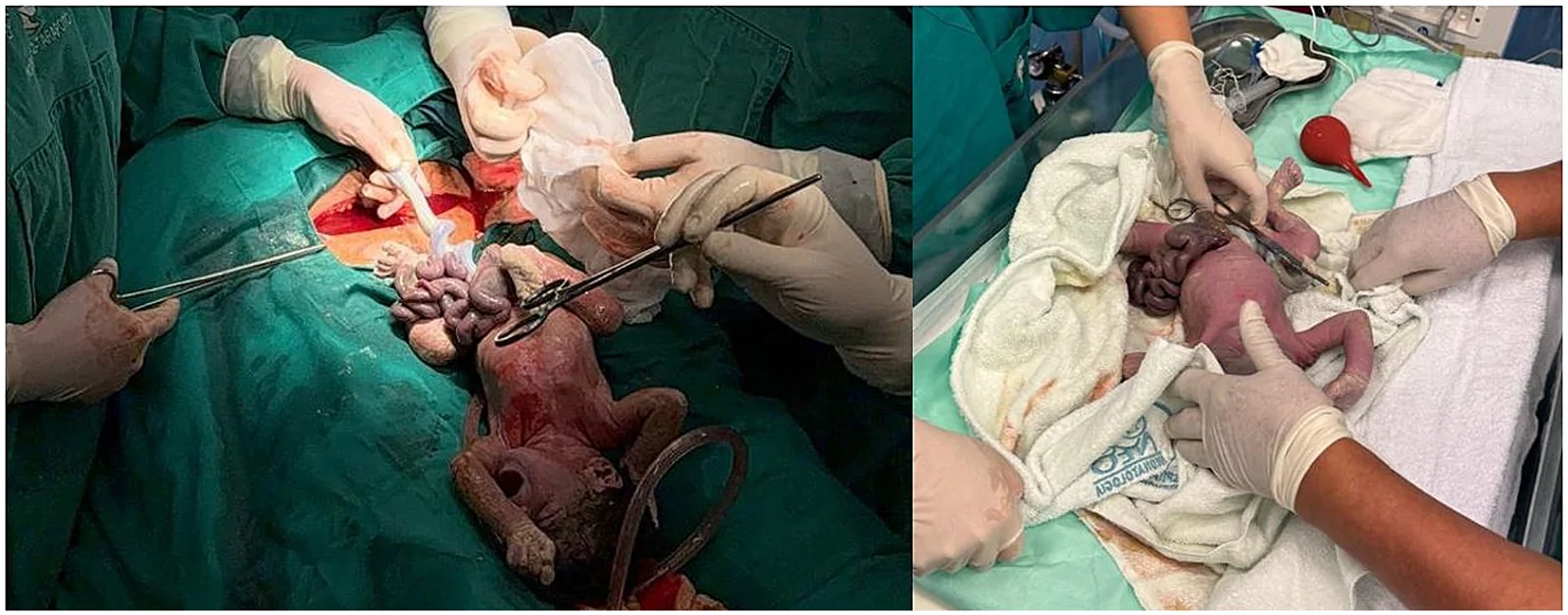
Intraoperative image obtained during cesarean delivery demonstrating the newborn with gastroschisis and exteriorized intestinal loops through a right paraumbilical abdominal wall defect.

Because of the abdominal wall defect and the condition of the exteriorized bowel, a silo was subsequently placed, followed by progressive reduction of the bowel and delayed abdominal closure. During subsequent surgical management, exploratory laparotomy was performed, with systematic assessment of the stomach, small intestine, and cecum. No intestinal atresia was identified. A progressive reduction in digestive contents was observed, suggestive of intestinal obstruction. Definitive abdominal wall closure was then performed with skin closure using a fine 4–0 suture. Following the procedure, transient hypoxemia developed, with oxygen saturation of 90%–91%, requiring postoperative oxygen therapy (Fig. 3).

**Figure 3:**
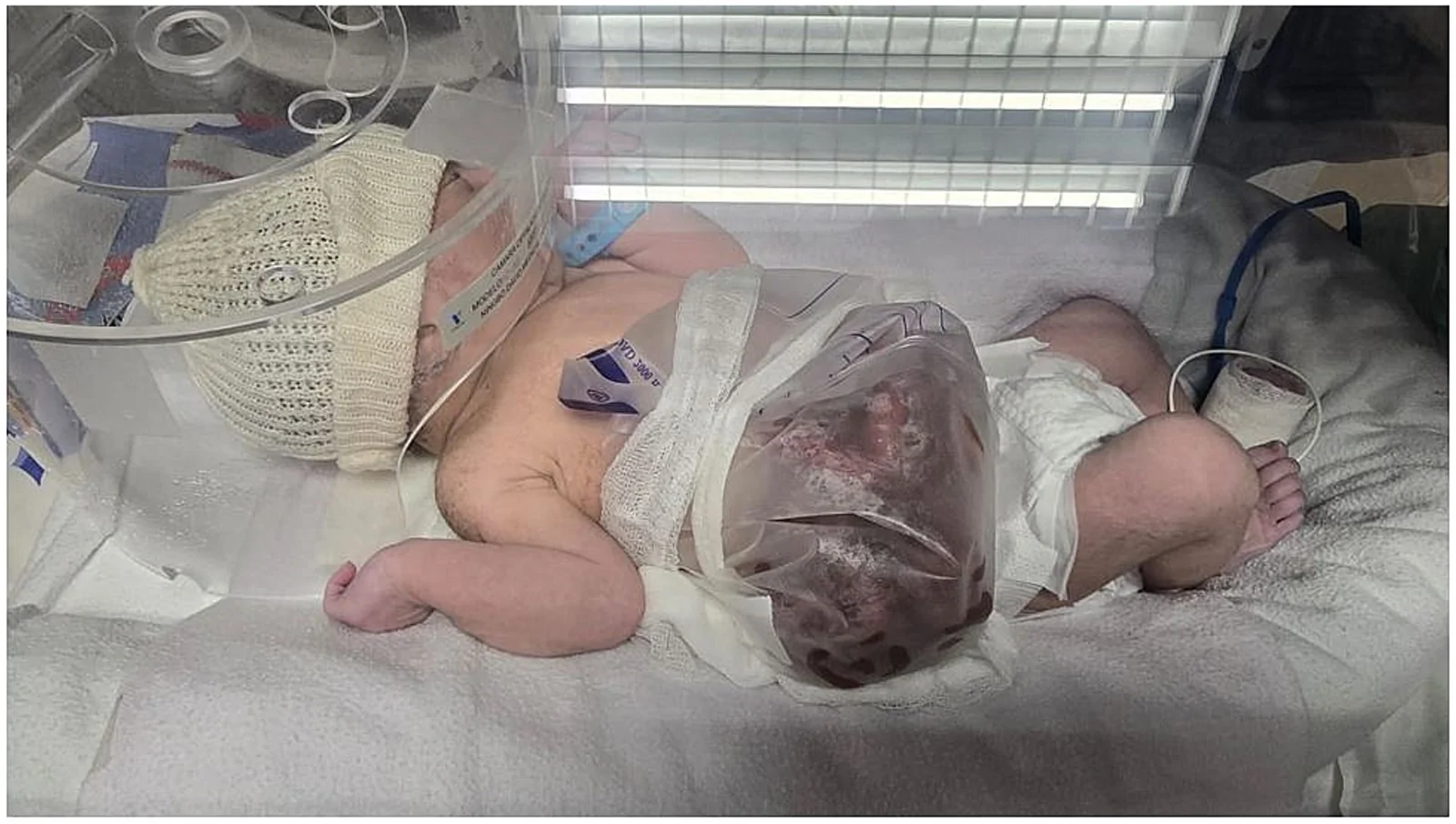
Surgical management of neonatal gastroschisis with silo placement and staged reduction of the exteriorized bowel loops prior to definitive abdominal wall closure.

During the neonatal course, echocardiography demonstrated a large PDA measuring 5 mm, associated with a systolic murmur best heard over the pulmonary area. The infant was in sinus rhythm with a heart rate of 145 beats/min. Pediatric cardiology recommended pharmacological closure with paracetamol at a dose of 15 mg/kg every 6 h for 5 days, together with strict fluid-volume control and maintenance of hemoglobin concentration above 12 g/dl. Serial echocardiographic evaluations were performed, and follow-up echocardiography demonstrated complete closure of the PDA.

Because of the risk of infection associated with prematurity, exposed bowel, and surgical intervention, empirical treatment with meropenem and vancomycin was initiated for suspected neonatal sepsis. Initial laboratory evaluation showed a C-reactive protein level of 6.70 mg/l, leukocyte count of 9.80 × 10^3^/μl, 55.7% segmented neutrophils, 0% immature neutrophils, and a platelet count of 241 × 10^3^/μl. The infant remained normothermic at 37.0°C. Blood cultures obtained on the day of birth remained negative after 5 days of incubation; urine and subsequent catheter-tip cultures were also negative. Erythromycin was administered as a prokinetic agent during the neonatal course.

Enteral feeding was introduced progressively according to clinical tolerance. The newborn subsequently showed favorable clinical evolution, with adequate oral tolerance, preserved rooting and sucking reflexes, and hemodynamic stability. After 28 days of hospitalization, she weighed 2005 g and was discharged with outpatient follow-up by pediatrics and pediatric surgery.

The mother had an uncomplicated immediate postpartum course, without hemorrhagic or infectious complications, and was discharged 2 days after the cesarean section.

## Discussion

Gastroschisis is a congenital abdominal wall defect associated with significant neonatal morbidity, particularly because of intestinal exposure, inflammation, edema, intestinal dysfunction, feeding difficulties, and the potential need for prolonged hospitalization and intensive neonatal care [1, 2]. Prenatal diagnosis plays an important role in the management of affected pregnancies because it allows fetal assessment, serial ultrasound surveillance, identification of sonographic markers associated with complex disease, delivery planning, and coordination of specialized neonatal and surgical care [1, 4, 7]. In the present case, prenatal evaluation identified gastroschisis and an associated true umbilical cord knot, while the subsequent decrease in fetal movements prompted further fetal assessment and recognition of fetal compromise.

Prenatal ultrasound is particularly relevant in gastroschisis because several sonographic findings may help identify fetuses at increased risk of complex disease and adverse neonatal outcomes [4, 7]. Although the initial ultrasound in our patient suggested intestinal atresia based on a “double bubble” appearance, subsequent specialized evaluation confirmed gastroschisis, and intestinal atresia was not identified during postnatal surgical exploration. This discrepancy illustrates that individual prenatal ultrasound findings should be interpreted within the complete clinical and sonographic context and subsequently correlated with postnatal findings [1, 4, 7].

The presence of a true umbilical cord knot represented an additional concern in this pregnancy. In our patient, decreased fetal movements were followed by a non-reactive fetal heart rate tracing with variable decelerations, indicating fetal compromise and leading to multidisciplinary obstetric assessment. Because of the fetal condition and persistent uterine contractions despite tocolytic therapy, emergency cesarean delivery was performed. Importantly, the indication for cesarean delivery in this case was based on evidence of fetal compromise and the obstetric circumstances rather than on the diagnosis of gastroschisis alone. The prenatal identification of gastroschisis nevertheless allowed the obstetric, neonatal, and pediatric surgical teams to anticipate the need for immediate specialized neonatal management [1, 4, 7].

Prematurity and low birth weight may further increase the complexity of neonatal management in infants with gastroschisis. The combination of prematurity with exposed abdominal viscera, respiratory vulnerability, feeding difficulties, and the need for surgical intervention may result in prolonged intensive care and hospitalization [1, 2]. Our patient was born at 33 weeks of gestation with a birth weight of 1850 g and required admission to the Neonatal Intensive Care Unit. Her management involved neonatology and pediatric surgery, with pediatric cardiology subsequently participating because of the development of a significant PDA.

Surgical management of gastroschisis should be individualized according to the condition of the newborn, the degree of intestinal edema, and the ability of the abdominal cavity to accommodate the exteriorized bowel [1, 3]. When primary closure is not feasible or may produce excessive intra-abdominal pressure, staged reduction with a silo followed by delayed abdominal closure may be used. In our patient, silo placement allowed progressive reduction of the exteriorized bowel before definitive abdominal closure. The favorable postoperative course supports the importance of individualized surgical management and close neonatal monitoring in premature infants with gastroschisis.

The coexistence of gastroschisis and PDA added another level of complexity to the clinical management of this preterm newborn. PDA is a frequent cardiovascular condition in premature infants and may influence pulmonary and systemic hemodynamics, making careful assessment of fluid status and cardiovascular function particularly important [8]. In our patient, echocardiography demonstrated a 5-mm PDA associated with a systolic murmur. Pediatric cardiology recommended pharmacological treatment with paracetamol, together with strict fluid-volume control and serial echocardiographic assessment. Complete closure of the PDA was subsequently documented. This multidisciplinary cardiovascular assessment allowed the PDA to be managed concurrently with the surgical and neonatal treatment of gastroschisis.

Feeding management represents another important challenge in newborns with gastroschisis. Intestinal inflammation, edema, dysmotility, prematurity, and postoperative gastrointestinal dysfunction may delay the establishment of adequate enteral nutrition [1, 3]. In our patient, enteral feeding was introduced progressively according to clinical tolerance. The newborn subsequently developed adequate oral tolerance, with preserved rooting and sucking reflexes, and was discharged after 28 days of hospitalization without documented necrotizing enterocolitis.

The patient also received empirical antibiotic therapy because of the clinical risk associated with prematurity, exposed bowel, and surgical intervention. Initial inflammatory and hematological parameters were monitored, while blood, urine, and catheter-tip cultures remained negative. This course illustrates the importance of continuous clinical reassessment in high-risk neonates and of correlating laboratory findings with the overall clinical condition when evaluating suspected neonatal infection.

Erythromycin was administered in our patient as a prokinetic agent in the context of feeding difficulties. However, its use should be interpreted as an individualized therapeutic intervention rather than as an established standard component of gastroschisis management. The available evidence regarding pharmacological stimulation of gastrointestinal motility in premature infants remains variable, and the potential benefits should be weighed against the limitations of the available evidence. In the present case, erythromycin was administered together with progressive enteral feeding and close clinical monitoring.

Overall, this case highlights the importance of prenatal care, early diagnosis, fetal surveillance, and coordinated multidisciplinary management in pregnancies complicated by gastroschisis. Prenatal identification of the abdominal wall defect allowed appropriate preparation for delivery and immediate neonatal surgical care, while the recognition of fetal compromise permitted timely obstetric intervention. After birth, coordinated management involving neonatology, pediatric surgery, and pediatric cardiology addressed the different clinical problems that emerged during hospitalization.

This case also emphasizes the educational importance of adequate counseling during prenatal care. Pregnant women should receive clear information regarding the importance of regular prenatal follow-up, fetal movement monitoring, warning signs requiring urgent assessment, and the potential need for delivery at a center capable of providing neonatal intensive and pediatric surgical care. For healthcare professionals, the case illustrates the importance of early communication and coordinated decision-making among obstetrics, maternal-fetal medicine, neonatology, pediatric surgery, and pediatric cardiology when gastroschisis and associated complications are identified.

The favorable outcome in this patient, despite prematurity, gastroschisis, PDA, suspected neonatal sepsis, and feeding difficulties, illustrates the potential value of coordinated multidisciplinary care in the management of complex neonatal conditions in a regional hospital setting.
